# Prevalence and Determinants of Tobacco Smoking Among University Students in Jordan: A Cross-Sectional Study

**DOI:** 10.1177/1179173X251377625

**Published:** 2025-09-26

**Authors:** Hana Taha, Ameen Al-Maayeh, Noora Al Momani, Lana al Natour, Shahid Abu Abboud, Abdel Rahman AlRamahi, Suhib Awamleh, Abdallah Al-Ani, Rania Ali Albsoul, Sireen M. Alkhaldi, Vanja Berggren

**Affiliations:** 1 Department of Family and Community Medicine, School of Medicine, 54658 The University of Jordan, Amman, Jordan; 2 Department of Neurobiology, Care Science and Society, 27106 Karolinska Institutet, Stockholm, Sweden; 3 Faculty of Medicine, 34419 The Hashemite University, Zarqa, Jordan; 4 Office of Scientific Affairs and Research, 37559 King Hussein Cancer Center, Amman, Jordan

**Keywords:** smoking, prevalence, university students, knowledge, attitudes, practices, Jordan

## Abstract

**Background:**

Jordan has one of the highest rates of tobacco smokers worldwide. This study aims to assess the prevalence and the determinants of tobacco smoking among university students in Jordan, including sociodemographic and cultural factors as well as knowledge and attitudes towards smoking.

**Methods:**

A cross-sectional study was conducted on a randomly selected sample of 763 university students from two public universities in Jordan (The Hashemite University and the University of Jordan) in 2024. The participants filled in a self-administered, structured paper questionnaire. The data was analyzed using descriptive and multivariate analysis by SPSS version 30. Statistical significance was set at *P* < .05 to assess the relationships between smoking behavior, sociodemographic factors, and various other variables.

**Results:**

Of the 763 university students who participated in this study, 561 participants (73.5%) were identified as smokers. Gender, age, and nationality were all significantly associated with smoking. However, GPA was inversely correlated with smoking, as the lowest smoking rate was among participants with a GPA between 3.5 and 4. Stress and the number of close friends who smoke were both identified as significant factors associated with smoking. Even though most participants agreed that smoking is a serious health hazard, this was not significantly associated with the intention to stop smoking.

**Conclusion:**

Our study revealed the widespread of smoking among Jordanian university students. We were able to identify multiple significant associations across sociodemographic, knowledge, and attitude factors. Targeted interventions in universities should prioritize smoking cessation programs, awareness campaigns, and academic stress management with a particular focus on addressing peer-driven smoking behaviors.

## Introduction

Smoking tobacco is a serious global public health problem and a significant cause of morbidity and mortality worldwide.^1^ The estimated global prevalence of smoking is 32.6% among men, 6.5% among women, and it ranges from 10 to 47.5% among youth and young adults in universities.^2^ Based on the 2023 global statistics of the World Health Organization (WHO), every year, tobacco kills an estimated 8 million smokers and 1.3 million non-smokers who are exposed to second-hand smoking.^3^ Moreover, it is forecasted that smoking will cause 310 million deaths over the next 28 years.^4^ Smoking is a risk factor for numerous diseases and various cancers, including a strong correlation with lung cancer.^5^ To address the tobacco epidemic, the WHO has implemented numerous prevention programs worldwide.^6^

Smoking is a rising global concern, particularly in low- and middle-income countries (LMIC).^7^ Around 80% of the world’s tobacco users live in LMIC. Based on the available literature, tobacco consumption increased in Africa and the Middle East by 57% from 1990 to 2009, while it decreased in Western countries by 26% during the same period.^8^ Middle Eastern countries demonstrated a predominance of male smokers, particularly cigarette smoking, which largely stems from social and cultural acceptability that reflects the difference in gender norms between males and females.^9^ However, Hookah smoking has gained popularity among Middle Eastern women, partly due to the increasing social acceptance and the misconception that it is harmless compared to cigarette smoking, due to its fruity and sweet flavors.^10^

While the initiation of smoking starts before the age of 18, young adulthood is the period during which smoking increases in frequency, develops into a regular habit, and ultimately, lead to nicotine dependence.^11^ Young adults, especially those in university settings, experience fundamental changes in social contexts and identity as they transition away from home and get introduced to a diverse set of peers.^12^ Their experienced and self-perceived degree of higher independence and new peers are postulated to augment smoking habits.^11,12^ Additionally, evidence exists that tobacco manufacturers specifically market to young adults.^13^ It is noteworthy to mention that even in nations where tobacco manufacturing is prohibited, such as Saudi Arabia, smoking rates are still concerning.^14^ Nasser et al, demonstrates that smoking rates across the entire Arab world are alarmingly high.^15^ However, waterpipe smoking rates have surged in the last two decades reflecting an even higher prevalence than cigarette smoking among university students. Such trends were observed across a number of Middle Eastern countries such as Syria, Jordan, Lebanon, and Palestine.^16^ These rates could be attributed to the availability of tobacco products, their low cost, and popularity in popular settings like cafes where young adults gather with their peers for entertainment.^16,17^

Various studies emphasized the impact of close relations on smoking habits, with siblings, friends, and especially parents playing a substantial role as role models. As children and young adults tend to imitate grown-ups, parental smoking becomes a crucial determinant of youth smoking habits.^18^ Having smoking best friends, positive attitudes towards smoking, and passive smoking in the house were identified as crucial risk factors for initiation of smoking.^19^ Male gender, lower academic performance and friends or family members who smoke were directly associated with smoking.^20,21^ Interestingly, while higher family income was associated to higher smoking rates in some reports, others found inverse associations between financial hardship and smoking rates.^2,22^

According to Jordan’s STEPwise non-communicable disease risk factor surveillance (STEPS) surveys, the proportion of males who smoke tobacco daily rose from 49.6% in 2007 to 58.0% in 2019, while the percentage of female daily smokers increased from 5.7% to 10.8% over the same period.^23^ In Jordan, tobacco consumption research is faint despite it having one of the highest smoking rates in the world. There is a lack of specific data about the prevalence and the determinants of smoking habits among university students in Jordan and interventions in Jordan. This gap highlights the need for more targeted research to provide evidence-based data for designing effective prevention programs and addressing the challenges and opportunities for tobacco control in Jordan. Hence, this study aims to assess the prevalence and the determinants of tobacco smoking among university students in Jordan, including sociodemographic and cultural factors as well as knowledge and attitudes towards smoking.

## Methods

### Study Setting

Jordan is a lower-middle-income country with a population of 11 million.^24^ The country’s literacy rate ranks higher than the global average, with 98% of adult Jordanians considered literate. The kingdom’s higher education system consists of two sectors: the public sector, which comprises 10 universities, and the private sector, which contains 18 universities.

This study was conducted between January and March 2024 at two public universities in Jordan: the Hashemite University (HU) and the University of Jordan (UJ). HU is located in Zarqa governorate with 14 faculties and almost 29 600 students, UJ is located in Amman with 20 faculties and almost 47 000 students.^25^

### Study Design

A quantitative descriptive cross-sectional study design was employed to investigate the prevalence, knowledge, attitudes, and determinants of smoking among university students in Jordan. The cross-sectional approach was appropriate for this study since it captures a precise snapshot at a particular point in time. Also, it is inexpensive, quick to implement, allows for the analysis of various variables, and provides baseline data for decision making and for further research.

### Ethical Considerations

Ethical approval for this study was obtained from the Hashemite University (HU) Institutional Review Board (IRB) (No.2300941/707). Before offering the questionnaire to the students, the researchers explained the study aim and process. Those who agreed to participate gave written consent. Anonymity of participants was ensured, and the data was confidential. Participants had the right to withdraw from the study without explanation at any point throughout the data collection.

### Study Population

Between January and March 2024, personal interviews were conducted with a random sample of 763 university students from both genders enrolled in UJ (396) and HU (367) to assess smoking prevalence, awareness, attitudes, and determinants. The minimum sample size was calculated using the EPI INFO STATCALC tool (version 7.2.6.0). At a 5% margin of error, 95% confidence level, and 50% response rate, the minimum required sample size was 382 participants. However, we recruited a random sample of 763 students to enhance the representation of the study population.

The data was collected in face-to-face interviews. The potential participant reviewed the research information page of the questionnaire, and the investigator answered any questions. Following that, those who agreed to participate in the study were asked to sign a written consent. The inclusion criteria were university student aged 18 or above and willing to complete the questionnaire. The exclusion criteria included university students younger than 18 years old and those not willing to complete the questionnaire.

### Measurement Tool

The data was collected from each participant individually using a structured questionnaire, which was developed based on existing literature,^26-29^ and validated by two public health experts from the School of Medicine at the University of Jordan. The questionnaire comprised five domains, including both multiple-choice and open-ended questions, that covered sociodemographic characteristics, smoking behavior, economic influences on smoking, psychosocial determinants of smoking status, health perceptions, and attitudes (Refer to supplemental material).

The demographic information domain included gender, age, nationality, faculty, academic year, academic performance (GPA), place of residence, monthly family income, number of family members, and marital status. The smoking behavior domain included smoking history, current smoking habits, age of initiation, types of tobacco used, attempts to quitting smoking, and smoking frequency. Smokers were categorized into “current smokers” and “non-smokers”. Current smokers were defined as those who smoked at least one form of tobacco/nicotine product once per day. Non-smokers were defined as those who do not consume any form of tobacco/nicotine products on daily, weekly, or monthly basis. Moreover, those who have abstained/quit smoking for more than 6 months, were also considered non-smokers.

Smoking type (i.e., cigarettes, waterpipe, vape, etc.) was determined based on whether participants have ‘regularly’ used that smoking modality in the past month. The economic and behavioral influences domain concerned itself with exploring willingness to pay certain threshold for smoking products, self-perceived behavioral changes to price changes, current level of physical activity, and smoking behaviors under different conditions, such as stress or social situations.

The psychosocial influences domain included social influences such as family and friends’ smoking habits, peer pressure, smoking in different scenarios, and the effect of stress, pleasure, and relief associated with smoking. Finally, the health perceptions and attitudes domain included attitudes towards smoking as a health hazard, knowledge of smoking risks, enjoyment of smoking, and perceptions of smoking’s impact on health, concentration, and alertness.

### Data Collection

The data was collected from January to March 2024 by using a paper self-administered structured questionnaire. For the aforementioned period, affiliated researchers visited the campuses of both UJ and HU. Participants were chosen at random throughout the campuses and were invited to complete a questionnaire concerned with smoking perceptions and habits. Participants were recruited from outside lecture halls, gathering spots, food courts, and dorms.

The researchers initiated the interaction by introducing themselves, clarifying the study objectives and relevance, while providing assurances regarding the confidentiality of participants’ input. Additionally, the researchers estimated the time required to complete the questionnaire. Subsequently, participants were respectfully asked about their inclination towards participation in the study, and those who agreed to participate gave written consent.

Respondents were invited to pose any inquiries while filling in the questionnaire, all of which were comprehensively addressed by the researcher. Upon finishing answering the questionnaire, the participants were thanked for their cooperation.

### Statistical Analysis

All data was analyzed using SPSS version 30.0.0. Descriptive statistics were utilized to showcase the data in the form of frequencies and percentages (n(%) for categorical variables and mean and standard deviations (mean ± SD) for continuous variables. Associations between categorical variables were explored using the Chi-squared test. In the case of small expected frequencies, the Fischer’s Exact Test was used. Variables measured on a 5-point Likert scale (e.g., perceptions and attitudes) were examined for differences in their means among categorical groups (i.e., current smokers and non-smokers) using the independent sample t-test. A *P*-value of less than 0.05 was considered statistically significant for all conducted analyses.

## Results

### Participants’ Sociodemographic Characteristics

A total of 763 participants were included in the study, primarily comprising 468 males (61.3%). The majority were single (739, 96.9%) and Jordanian (726, 95.2%). Participants were categorized into four age groups: 18-20 years, 20-22 years, 22-24 years, and above 25 years, with the largest proportion falling within the 20-22 age range (51.4%). In terms of academic performance, the participants who had a GPA classified as Good (2.5-3.0; 34.7%) or Very Good (3.0-3.5; 30.2%). A significant majority resided in the central region of Jordan (88.6%). Regarding faculty distribution, participants were mainly from human studies faculties (49.7%), followed by science (28.7%) and health studies (20.3%). More than half of the respondents were in their mid-years of study (years 3-4; 55.4%), while 36.3% were in their early years (years 1-2). Income levels varied, with 36.2% of participants reporting a lower income, followed by 26.1% in the lower-middle-income category and 22.8% in the high-income category.

Of the total sample, 561 participants (73.5%) identified as smokers, while 202 (26.5%) were non-smokers. Gender was significantly associated with smoking status, as 85% of males were current smokers compared to 55.3% of females (*P* < .001). Age also showed a significant association, with the highest smoking prevalence observed among participants aged 22-24 years (86%) and the lowest among those aged 18-20 years (68.5%) (*P* = .015). Nationality was a significant factor, with 74.2% of Jordanians reporting smoking compared to 59.5% of non-Jordanians (*P* = .047). In contrast, marital status, faculty, and place of residence were not significantly associated with smoking status.

Academic performance, measured by GPA, was inversely associated with smoking prevalence. Students with a GPA below 2.0 had the highest smoking rate (85.7%), while those with a GPA between 3.5 and 4.0 had the lowest (69.1%) (*P* = .01). The year of study was also significantly associated with smoking behavior; students in advanced years (5-8) reported a higher smoking rate (88.9%) compared to those in early years (1-2) (68.6%) (*P* = .004). Additionally, monthly income was associated with smoking status, with higher income levels corresponding to greater smoking prevalence (*P* = .038). An overview of these sociodemographic characteristics and their associations with smoking status is provided in Table 1.

**Table 1.** Sociodemographic Characteristics of Participants and Their Association With Smoking Status

Variable	Category	Total	N (Smokers)	% (Smokers)	X^2^	*P*-value
Gender	Male	468	398	85.0%	82.49	<0.001
	Female	295	163	55.3%		
Age	18-20	251	172	68.5%	12.31	0.015
	20-22	392	288	73.5%		
	22-24	100	86	86.0%		
	>25	20	15	75.0%		
Nationality	Jordanian	726	539	74.2%	3.95	0.047
	Other	37	22	59.5%		
Marital status	Single	739	543	73.5%	--	0.310
	Married	16	11	68.8%		
	Other	8	7	87.5%		
Faculty	Health studies faculties	155	106	68.4%	4.394	0.220
	Human studies faculties	379	284	75.0%		
	Science studies faculties	219	166	75.8%		
	Graduate studies faculties	10	5	50.0%		
GPA	<2	35	30	85.7%	13.65	0.010
	2-2.5	138	109	79.0%		
	2.5-3	265	204	77.0%		
	3-3.5	231	153	66.2%		
	3.5-4	94	65	69.1%		
Year of study	Early years (1-2)	277	190	68.6%	11.30	0.004
	Mid years (3-4)	423	315	74.5%		
	Advanced years (5-8)	63	56	88.9%		
Residence	Central region	676	493	72.9%	--	0.490
	Northern region	78	60	76.9%		
	Southern region	9	8	88.9%		
Monthly income	Lower income	276	187	67.8%	8.43	0.038
	Lower-middle income	199	148	74.4%		
	Upper-middle income	114	89	78.1%		
	High income	174	137	78.7%		

**Figure 1.**
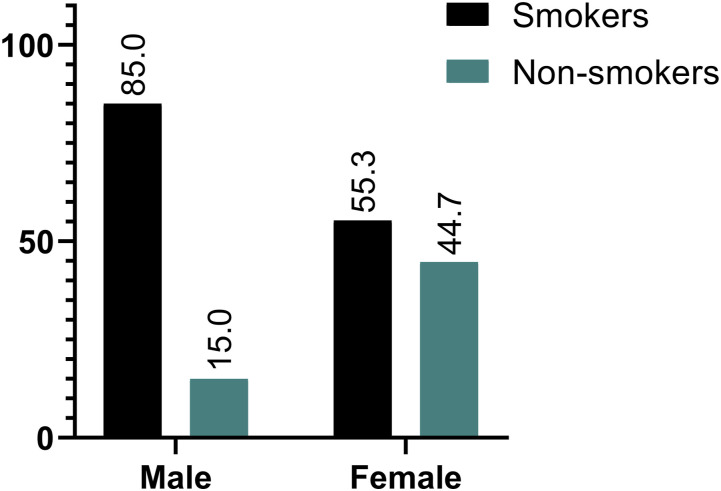
Smoking Status Stratified per Gender

We found that males heavily predominated the gender distribution, as we found that out of 561 smokers included in the sample, 71% of them were male, as shown in Figure 1.

### Smoking Behavior and Economic Factors

Between current smokers, more than half (55.6%) stated smoking less than one pack per day, 37.1% smoked between one and two packs daily, and 7.3% consumed more than two packs per day. Concerning the type of smoking, 34.4% used mixed types, 26.4% smoked only cigarettes, 22.6% used only vape, and 16.5% smoked only hookah. Of the 263 mixed types users, 79.8% were current smokers. Similarly, of the 202 and 173 exclusively cigarette and vape users, 87.1% and 70.5% are current smokers, respectively. Only 41.3% of 126 exclusive waterpipe/hookah users were current smokers.

Former attempts to quit smoking were reported by 59.9% of smokers, while 40.1% had not attempted to quit. The starting age for smoking was diverse, with 39% reported starting between 16 and 18 years, 22.6% after 18 years, and 15.4% between 12 and 14 years.

In terms of weekly costs of cigarettes, 44.7% spent less than 10 JD (≈14.1 USD), 31.8% spent between 10 and 20 JD (≈14.1 – 28.21 USD), 17% spent 20 and 30 JD (≈28.21 – 42.31 USD), and 6.5% spent 30 and 40 JD (≈42.31 – 56.42 USD). When asked about the possible effect of a price doubling, 40.6% showed that they would continue smoking the same kind, 39% would smoke less, 15.2% would switch to a cheaper alternative, and 5.2% would smoke more.

Physical activity levels among the participants were diverse, 28.7% reported no physical activity, 14 8% engaged in daily activity, and the remaining respondents reported engaging in physical activity between one and six days per week. Everyday situations associated with smoking included social situations with friends (63.8%), after eating (59.1%), during stressful situations (49.3%), and routinely throughout the day (42.5%) as shown in Table 2.

**Table 2.** Smoking Behavior and Economic Influences

Variable	Category	N	%
Packs per day	Less than 1	424	55.6%
	1 to 2	283	37.1%
	More than 2	56	7.3%
Type of smoking	Mixed types	262	34.4%
	Only cigarette	202	26.4%
	Only hookah	126	16.5%
	Only vape	173	22.6%
Previous attempts to quit smoking	Yes	457	59.9%
	No	306	40.1%
First cigarette age	Less than 10	35	4.6%
	10 to 12	27	3.5%
	12 to 14	118	15.4%
	14 to 16	113	14.8%
	16 to 18	297	39.0%
	More than 18	173	22.6%
Price of cigarettes per week*	Less than 10 JD	341	44.7%
	10-20 JD	242	31.8%
	20-30 JD	130	17.0%
	30-40 JD	50	6.5%
	More than 40 JD	0	0.0%
Doubling of price	Smoke less	297	39.0%
	Smoke a cheaper alternative	116	15.2%
	Smoke the same kind	310	40.6%
	Smoke more	40	5.2%
Physical activity	None	219	28.7%
	1 day per week	56	7.3%
	2 days per week	83	10.8%
	3 days per week	103	13.5%
	4 days per week	97	12.7%
	5 days per week	75	9.8%
	6 days per week	17	2.2%
	Every day	113	14.8%
Smoking scenarios	After eating	452	59.1%
	Social scenarios with friends	488	63.8%
	Stressful scenarios	377	49.3%
	Routinely throughout the day	325	42.5%

*1 JD is equivalent to 1.41 USD.

### Influence of Psychosocial Factors on Smoking Status

A significant association was found between the belief in quitting smoking and smoking status (*P* < .001), with smokers showing the highest prevalence among those who reported a low belief in quitting (83.2%). The impact of stress on smoking behavior was also significantly associated with smoking (*P* = .03), with higher smoking rates observed among those reporting high stress (76.1%).

Factors such as the impact of pleasure on smoking behavior and perceived relief from smoking both showed significant associations. Participants who reported higher pleasure or relief from smoking had the highest prevalence of smoking, 79.3% and 83.2%, respectively, both with (*P* < .001). A positive association was also found between smoking and the number of close friends who smoked, with 76.3% of smokers reporting 3-4 friends who smoked (*P* < .001). A significant association was found between study concentration during smoking and smoking status (*P* < .001).

Factors such as willingness to accept help to quit smoking and smoking in the family (father, mother, siblings) did not show a statistically significant association with smoking status, as demonstrated in Table 3.

**Table 3.** Association of Psychosocial Factors With Smoking Status

Variable	Category	Total	N (Smokers)	% (Smokers)	X^2^	*P*-value
Belief in quitting smoking is possible	Low	201	168	83.2%	24.89	<0.001
	Medium	184	145	78.8%		
	High	378	248	65.4%		
Willingness to accept help to quit smoking	Low	304	231	75.7%	2.78	0.250
	Medium	123	93	75.6%		
	High	336	237	70.3%		
Impact of stress on smoking behavior	Low	197	143	72.6%	6.86	0.030
	Medium	119	77	64.2%		
	High	447	341	76.1%		
Impact of pleasure on smoking behavior	Low	187	121	64.7%	17.20	<0.001
	Medium	161	110	67.9%		
	High	415	330	79.3%		
Perceived relief from smoking	Low	190	112	58.9%	42.60	<0.001
	Medium	187	127	67.6%		
	High	386	322	83.2%		
Study concentration during smoking	Low	283	163	57.4%	65.64	<0.001
	Medium	131	98	74.8%		
	High	349	300	86.0%		
Smoking in family	Father	466	351	75.3%	2.86	0.230
	Mother	166	121	72.8%	0.21	0.880
	Siblings	390	281	72.1%	1.33	0.510
Smoking among close friends	None	21	12	57.1%	16.29	<0.001
	1-2	113	68	58.6%		
	3-4	629	481	76.3%		

### Perception and Attitude towards Smoking

Most participants agreed that smoking is a serious health hazard (Mean = 4.15, SD = 1.293). Perceptions of hazard did not significantly differ between current smokers and non-smokers (MD: 0.097; 95%CI: −0.110 – 0.305). Current smokers were significantly more likely to report higher perceptions of smoking enjoyment (MD: −0.571; 95%CI: −0.773 to −0.369) and pleasure (MD: −0.791; 95%CI: −1.020 to −0.561). Moreover, current smokers were significantly more likely to report that smoking helps with concentration (MD: −1.119; 95%CI: −1.351 to −0.886), provide alertness (MD: −0.850; 95%CI: −1.072 to −0.628), and reduces fatigue (MD: −0.701; 95%CI: −0.933 to −0.470).

Furthermore, compared to non-smokers, current smokers were significantly more likely to smoke under stress (MD: −1.214; 95%CI: −1.443 to −0.985), have strong craving when abstaining for short periods (MD: −0.837; 95%CI: −1.063 to −0.611), and feel discomfort when running out of cigarettes (MD: −1.181; 95%CI: −1.418 to −0.945). Interestingly, social smoking was normalized across the cohort (Mean = 3.62, SD = 1.379). Similarly, there is consensus that smoking is considered part of the Jordanian cultural traditions (Mean = 3.32, SD = 1.569).

Finally, the majority of the cohort disagreed that smoking is associated with attractiveness (Mean = 1.90, SD = 1.362) or maturity (Mean = 2.29, SD = 1.416). These perceptions did not significant differ between current and non-smokers. Additionally, both current and non-smokers believe that smoking does harm even when consumed in small amounts (Mean = 2.73, SD = 1.528) as presented in Table 4.

**Table 4.** Perceptions and Attitudes Towards Smoking

Perceptions and attitudes	Overall Mean^a^	SD	*P*-value	Mean Difference^b^	Lower 95%CI	Upper 95%CI
Smoking is a serious health hazard	4.15	1.293	.359	0.097	−.110	.305
Enjoyment level of cigarette smoking	3.76	1.283	**<.001**	−0.571	−.773	−.369
Feeling more mature and Sophisticated when smoking	2.29	1.416	.061	−0.217	−.444	.010
Feeling more attracted to the opposite sex when smoking	1.90	1.362	.53	−0.070	−0.289	0.149
Definite pleasure when smoking	3.18	1.470	**<.001**	−0.791	−1.020	−0.561
Smoking more when worried	3.64	1.523	**<.001**	−1.214	−1.443	−0.985
Feeling lift and alert when smoking	3.15	1.434	**<.001**	−0.850	−1.072	−0.628
Smoking is perceived to help alleviate fatigue	2.66	1.476	**<.001**	−0.701	−0.933	−0.470
Smoking is perceived to aid in thinking and concentration	3.18	1.529	**<.001**	−1.119	−1.351	−0.886
Running out of cigarettes is perceived to cause significant discomfort	2.96	1.564	**<.001**	−1.181	−1.418	−0.945
The craving for cigarettes is perceived to be strong when forced to stop smoking for a period	3.39	1.451	**<.001**	−0.837	−1.063	−0.611
Smoking is perceived as a part of cultural traditions	3.32	1.569	.051	−0.251	−0.503	0.001
Smoking is considered to have minimal harm when consumed in small amounts	2.73	1.528	.609	0.064	−0.182	0.310
Smoking is perceived as socially acceptable	3.62	1.379	.653	−0.051	−0.273	0.171

^a^The overall mean represents the average responses for both current smokers and non-smokers.

^b^Mean differences refer to differences in responses between current smokers and non-smokers.

## Discussion

This cross-sectional study was conducted in the Hashemite Kingdom of Jordan to identify sociodemographic, economic, and psychological factors that influence the tendency to smoke among university students and their impact on the students’ attitudes towards smoking. Our study revealed that 73.5% of participants were smokers, which is alarmingly high compared to previous studies.^23,30^

The prevalence of smoking in our study is relatively higher among Jordanian university students for both males and females in comparison a 2014 report (i.e., 54.3% for males, 11.1% for females) and a 2008 report (i.e., 61.3% for males, 55.3% for females).^23,30^ These findings, in fact, are not surprising as Jordan its already known for its high rates of tobacco consumption.^31^ On the global level, it is one of six countries where tobacco use continues to grow despite the general trend of declining prevalence.^6^ Alkhatib et al, estimates the total cost attributable to smoking and secondhand smoke at 2018 million dollars in 2019, representing 4.7% of Jordan’s gross domestic product.^32^

While the increase could be attributed to methodological differences between the already scarce Jordanian reports, it could be a sign that national tobacco control efforts are either ineffective or undermined. Jordan’s Public Health Law number 47 of 2008 prohibits smoking in public areas and bans tobacco advertising. Yet, these laws are lax and lack any form of real enforcement. From a social perspective, smoking is deeply rooted in the Jordanian culture. While an exact phenomological analysis is yet to be produced, an analysis of the Jordanian tobacco market shows that individuals, irrespective of gender, are willing to spend 2.13 to 2.15 JDs per pack while earning less than 250 JDs per month.^33^ The Economics of Smoking in Jordan report indicates that more than 13.5% of the average Jordanian household income is spent on tobacco products.^34^ These numbers only serve to showcase the extent of addiction exhibited by the Jordanian public towards smoking. Moreover, these observations validate the finding that higher monthly income is associated with higher odds of smoking.

The burden of smoking being higher in males than females is also expected.^35^ Hossain et al, demonstrated that cultural and social factors enable males to engage with smoking relatively easier than females.^35^ Moreover, reports showcase that smoking is deeply associated with gender roles, particularly, the masculine construct.^36^ Similarly, smoking is generally associated with hegemonic masculinity, which refers to any conceptualization of men who typically promote being powerful, violent, risk takers, and traditionally reject feminine characteristics.^37^ Irrespective of the discrepancy between gender, the rates of smoking among Jordanian women are also seriously high. Haddad et al, believes that “the main advantage of smoking for males was calming down, while for females it was independence”.^38^ While women smoking conventional cigarettes is looked down upon within the Jordanian society, waterpipe smoking enjoys greater social acceptability and is a less stigmatized entry to further tobacco usage among women.

Our findings showed that the most common age for starting smoking was between 22 and 24 years old, which was against other reported studies which showed that university students often start early during their undergraduate years.^16^ Contrary to our findings, a longitudinal study conducted in 2011 found that starting smoking under the age of 15 was the strongest factor predicting nicotine dependence.^19,39^

Khader and Alsadi (2008) showed that type of undergraduate school could be a predictor for stress and smoking.^23^ They found that “students from the faculty of religion and law were less likely to smoke compared with students who attended other faculties”. These findings are contrary to our results which demonstrate no association between type of school/faculty and smoking prevalence. However, differences among different categories of school might have been underestimated due to the limited statistical power and representation of different faculties. In terms of academic performance, our analysis showed that most of the students who smoked had a GPA in the range between 2.0 and 2.5, within which smoking prevalence was 85.7%. These findings are consistent with Alkhalaf et al, which showed that all types of nicotine-containing products were found to be associated with poor academic achievement and worsening of the academic performance, including lower GPA and higher absenteeism rate.^40^

In terms of psycho-social factors, studies have pointed out that smokers have close relations with other smokers, such as siblings, friends, and especially parents. Our study revealed that there is a strong association between smoking and the number of close friends who smoked, in which 76.3% of smokers reported they had 3 to 4 close friends who were smokers. Number of friends who smoke was observed as the most common risk factor linked to cigarette use in terms of both initiation and continuation; the impact of peer pressure is also strongly documented for abuse of a variety of substances including cannabis and alcohol.^41,42^

On the other hand, across our cohort, parental smoking and number of smokers in the family members didn’t show any association with smoking behavior and nicotine dependence. This finding might appear contradictory to the greater body of literature; however, Waa et al, provides an alternative explanation of these findings.^43^ They postulate that parental smoking may not result in increasing the susceptibility of smoking among children but rather other parent-related determinants. These determinants include behaviors related to smoking socialization, parental expectations towards smoking, or even general parenting styles (e.g., degree of support, provision of pocket money). Interestingly, a Scottish cohort demonstrated that the very act of parental smoking has a significant effect before adolescence.^44^ In the case of our cohort, most participants started mid their undergraduate journey.

Finally, we have demonstrated cognitive dissonance among our cohort of current smokers. Despite acknowledging the hazardous risk of smoking, even when consumed in small amounts, smokers in our cohort associated the act with pleasure, relief, and perceived withdrawal symptoms, such as stress and worry, when away from smoking. While psychological stress is deemed as a primary driver of smoking initiation, these cognitive signs could be a sign of substance use disorder.^45^ These speculations could explain that despite a high intent to quit smoking, a large percentage of the present cohort failed.

### Limitations

Our results should be interpreted within the context of a number of limitations. The study employs a cross-sectional design within a very specific time frame. This could’ve resulted in missing responses from other sects of students not present within the cross-section. The design also limits our ability to pinpoint causative relationships between risk factors, sociodemographic characteristics, and smoking habits. Moreover, responses, due to resource availability, were extracted from two public universities only. Perceptions towards smoking and habits could be different within private institutions or other academic establishment representing the Jordanian student population away from the center. Due to the sample size and the varying sizes of faculties, certain groups may be under- or overrepresented, introducing potential selection bias. Another source of bias is that nicotine itself can act as a stressor^46^; consequently, smokers might report elevated levels of perceived stress that they mistakenly attribute to university pressures or their everyday activities.

## Conclusion

We demonstrated an alarmingly high smoking prevalence among Jordanian university students. Such prevalence was significantly associated with a number of sociodemographic factors including male gender, older age, lower academic performance, and higher monthly income. From a psycho-social perspective, smokers were able to realize that smoking was a health hazard even in minute amounts; yet, they associate it with pleasure, relief, and perceive withdrawal symptoms such as stress and worry. These habits are augmented by the presence of like-minded peers, all of which highlights the serious role of social reinforcement in smoking initiation and continuation.

## Supplemental Material

Supplemental Material - Prevalence and Determinants of Tobacco Smoking Among University Students in Jordan: A Cross-Sectional Study Supplemental Material for Prevalence and Determinants of Tobacco Smoking Among University Students in Jordan: A Cross-Sectional Study by Hana Taha, Ameen Al-Maayeh, Noora Al Momani, Lana al Natour, Shahid Abu Abboud, Abdel Rahman AlRamahi, Suhib Awamleh, Abdallah Al-Ani, Rania Ali Albsoul, Sireen M. Alkhaldi, Vanja Berggren in Tobacco Use Insights

## Data Availability

The datasets used and analyzed during the current study are available from the corresponding author upon request.*
