# Supplementary material for: Prevalence and Determinants of Tobacco Smoking Among University Students in Jordan: A Cross-Sectional Study
Source: Tob Use Insights. 2025 Sep 26;18:1179173X251377625. doi: 10.1177/1179173X251377625 (PMC12475322; doi:10.1177/1179173X251377625)
Supplement: Supplemental Material - Prevalence and Determinants of Tobacco Smoking Among University Students in Jordan: A Cross-Sectional Study [file sj-pdf-1-tui-10.1177_1179173X251377625.pdf]

Prevalence and determinants of tobacco smoking among university students in Jordan: A cross-sectional study

Questionnaire

Part A

1) Specify your gender
   a) Male
   b) Female
2) Choose the group that best resembles your age
   a) <18 years
   b) 18 – 20 years
   c) 20 – 22 years
   d) 22 – 24 years
   e) >25
3) Specify your nationality
   a) Jordanian
   b) Non-Jordanian
4) Specify your faculty of study
   a) Health studies
   b) Human studies (arts and humanities)
   c) Science studies
   d) Graduate studies
5) Specify your current year of study
   a) [Open-ended]
6) Choose the group that best resembles your GPA
   a) <2.0
   b) 2.0 – 2.5
   c) 2.5 – 3.0
   d) 3.0 – 3.5
   e) 3.5 – 4.0
7) Specify your current location of residence
   a) Amman
   b) Irbid
   c) Zarqa
   d) Mafraq
   e) Ajloun
   f) Jerash
   g) Madaba
   h) Balqa
   i) Karak
   j) Tafilieh
   k) Maan
8) Monthly income
   a) Lower income
   b) Lower – Middle income
   c) Middle – Upper income
   d) High income
9) Specify your current marital status
   a) Single

b) Married
c) Divorced
d) Widowed

10) Specify the current number of your family members
    a) [Open-ended]

Part B

1) Have you smoked at least one form of tobacco/nicotine product at least once on daily basis?
    a) Y/N
2) How many packs per day do you consume?
    a) [Open-ended]
3) Do you smoke cigarettes
    a) Y/N
4) Do you smoke waterpipe/hookah
    a) Y/N
5) Do you smoke E-cigarettes/Vape
    a) Y/N
6) In the past month, did you smoke cigarettes regularly
    a) Y/N
7) In the past month, did you smoke waterpipe/hookah regularly
    a) Y/N
8) In the past month, did you smoke E-cigarettes/Vape regularly
    a) Y/N
9) At what age did you start smoking?
    a) Less than 10 years
    b) 10 to 12 years
    c) 12 to 14 years
    d) 14 to 16 years
    e) 16 to 18 years
    f) More than 18 years
10) Have you considered quitting from smoking
    a) Y/N
11) For how long were you able to abstain from smoking
    a) [Open-ended]
12) Do you believe that quitting smoking is possible
    a) 5-point likert scale
13) Are you willing to accept help to quit smoking
    a) 5-point likert scale
14) Do you smoke in response to stress
    a) 5-point likert scale
15) Do you smoke to experience pleasure
    a) 5-point likert scale
16) Do you smoke to feel relieved
    a) 5-point likert scale
17) Do you smoke to feel concentrated for your studies
    a) 5-point likert scale
18) Do you smoke because your friends smoke
    a) 5-point likert scale
19) What percentage of your social network are smokers

a) Less than 20%
b) 20 – 40%
c) 40 – 80%
d) More than 80%

20) Is your father a smoker?
a) Y/N

21) Is your mother a smoker?
a) Y/N

22) Are any of your siblings smokers?
a) Y/N

23) Amount of money spent on cigarettes per week
a) Less than 10 JDs
b) 10 to 20 JDs
c) 20 to 30 JDs
d) 30 to 40 JDs

24) What is your reaction if the prices were doubled
a) Smoke less
b) Smoke a cheaper alternative
c) Smoke the same kind
d) Smoke more

25) When do you often smoke
a) After eating
b) Social gatherings with friends
c) During or after stressful scenarios
d) Routinely throughout the day

26) On how many days per week do you engage in strenuous physical activity
a) Zero
b) 1 day per week
c) 2 days per week
d) 3 days per week
e) 4 days per week
f) 5 days per week
g) 6 days per week
h) 7 days per week

Part C [All items are answered on a 5-item likert scale]

27) Do you believe that smoking is a health hazard
28) Do you believe that smoking enhances enjoyment levels
29) Do you believe that smoking makes more mature or sophisticated
30) Do you believe that smoking makes attractive
31) Do you believe that smoking gives definite pleasure
32) Do you smoke more when worried
33) Do you smoke more when in need to be alert
34) Do you smoke more when tired and in need to keep going
35) Do you smoke more when in need of thinking and concentration
36) Do you worry and experience discomfort if you ran out of cigarettes
37) Do you experience cravings when stopping smoking
38) Do you believe that smoking is part of our culture and traditions
39) Do you believe that smoking isn’t harmful in small doses
40) Do you believe that smoking is socially acceptable
